# Mitochondrial dysfunction and cellular senescence drive accelerated gestational aging in spontaneous preterm birth: a narrative review

**DOI:** 10.1007/s10495-026-02394-z

**Published:** 2026-07-08

**Authors:** Gagana Hanumaiah, Thejesh Srinivas, Akhila Vasudeva

**Affiliations:** 1 Department of Obstetrics and Gynaecology, Kasturba Medical College, Manipal Academy of Higher Education, Manipal, India; 2 Department of Critical Care Medicine, Kasturba Medical College, Manipal Academy of Higher Education, Manipal, India; 3 Division of Fetal Medicine, Department of Obstetrics and Gynaecology, Kasturba Medical College, Manipal Academy of Higher Education, Manipal, Karnataka 576104 India

**Keywords:** Preterm birth, Mitochondrial dysfunction, Cellular senescence, Ferroptosis, Inflammasome, Extracellular vesicles, Gestational aging

## Abstract

Spontaneous preterm birth (sPTB) is a leading cause of neonatal mortality and long-term morbidity worldwide, affecting approximately 15 million infants annually. Despite advances in obstetric care, its incidence has remained largely unchanged, reflecting an incomplete understanding of the biological mechanisms governing the timing of parturition. While infection and inflammation have traditionally dominated etiological models, these alone do not fully explain the heterogeneity in disease onset, progression, and outcomes. Emerging evidence suggests that sPTB may represent a state of accelerated gestational aging, in which cellular stress pathways prematurely activate labour mechanisms that are normally tightly regulated at term. This narrative review synthesizes current evidence linking mitochondrial dysfunction, oxidative stress, and cellular senescence to the pathogenesis of sPTB. Across gestational tissues, including the placenta, fetal membranes, decidua, cervix, and myometrium, molecular stress induces shifts characterized by impaired mitochondrial oxidative phosphorylation, increased reactive oxygen species generation, and activation of senescence-associated pathways. Beyond reflecting cellular injury, these processes actively propagate inflammatory signalling, extracellular matrix remodelling, and endocrine activation that collectively promote premature labour. We further integrate underexplored mechanistic pathways, including mitochondrial dynamics and mitophagy, ferroptosis, nicotinamide adenine dinucleotide (NAD⁺) metabolism, inflammasome activation, extracellular vesicle signalling, and deoxyribonucleic acid (DNA) damage responses. These interconnected pathways interact through damage-associated molecular patterns (DAMPs), cytokines, and extracellular vesicles to coordinate pathological crosstalk across the maternal-fetal interface. Emerging multi-marker biomarker strategies and targeted therapeutic approaches, including mitochondrial antioxidants, senolytic agents, and inflammasome inhibitors, are also discussed within this framework. Mitochondrial dysfunction and cellular senescence represent central biological axes linking molecular stress with premature activation of labour pathways in sPTB. Conceptualizing sPTB as accelerated gestational aging provides a unifying framework for integrating diverse mechanistic pathways, refining risk stratification, and guiding the development of targeted, precision-based interventions to reduce the global burden of prematurity.

## Introduction

Preterm birth, defined as delivery before 37 completed weeks of gestation, affects approximately 10% of all live births globally and represents the leading cause of neonatal mortality and childhood morbidity [1]. Spontaneous preterm birth (sPTB), accounting for two-thirds of all preterm deliveries, occurs through two primary pathways like spontaneous preterm labour with intact membranes and preterm premature rupture of membranes (PPROM) [2]. Survived preterm neonates face increased risks of respiratory distress syndrome, intraventricular haemorrhage, necrotizing enterocolitis, and long-term neurodevelopmental impairment, imposing substantial burdens on healthcare systems and families [3]. Despite improvements in neonatal intensive care, the incidence of sPTB has remained largely unchanged over recent decades, underscoring the need for novel mechanistic insights and preventive strategies [4].

Historically, intrauterine infection and inflammation dominated etiological models of sPTB, supported by evidence of microbial invasion and elevated inflammatory cytokines in many early preterm births [5]. However, a substantial proportion of sPTB cases occur without detectable infection, indicating that additional biological mechanisms contribute to premature labour activation [6]. This heterogeneity led to the concept of the “preterm parturition syndrome,” recognizing sPTB as a multifactorial condition with diverse pathways converging on common labour mechanisms [7]. Recent advances in reproductive biology and geroscience have introduced a paradigm shift by viewing pregnancy as a regulated process of biological aging within gestational tissues, with sPTB representing accelerated gestational aging [8, 9].

**Fig. 1:**
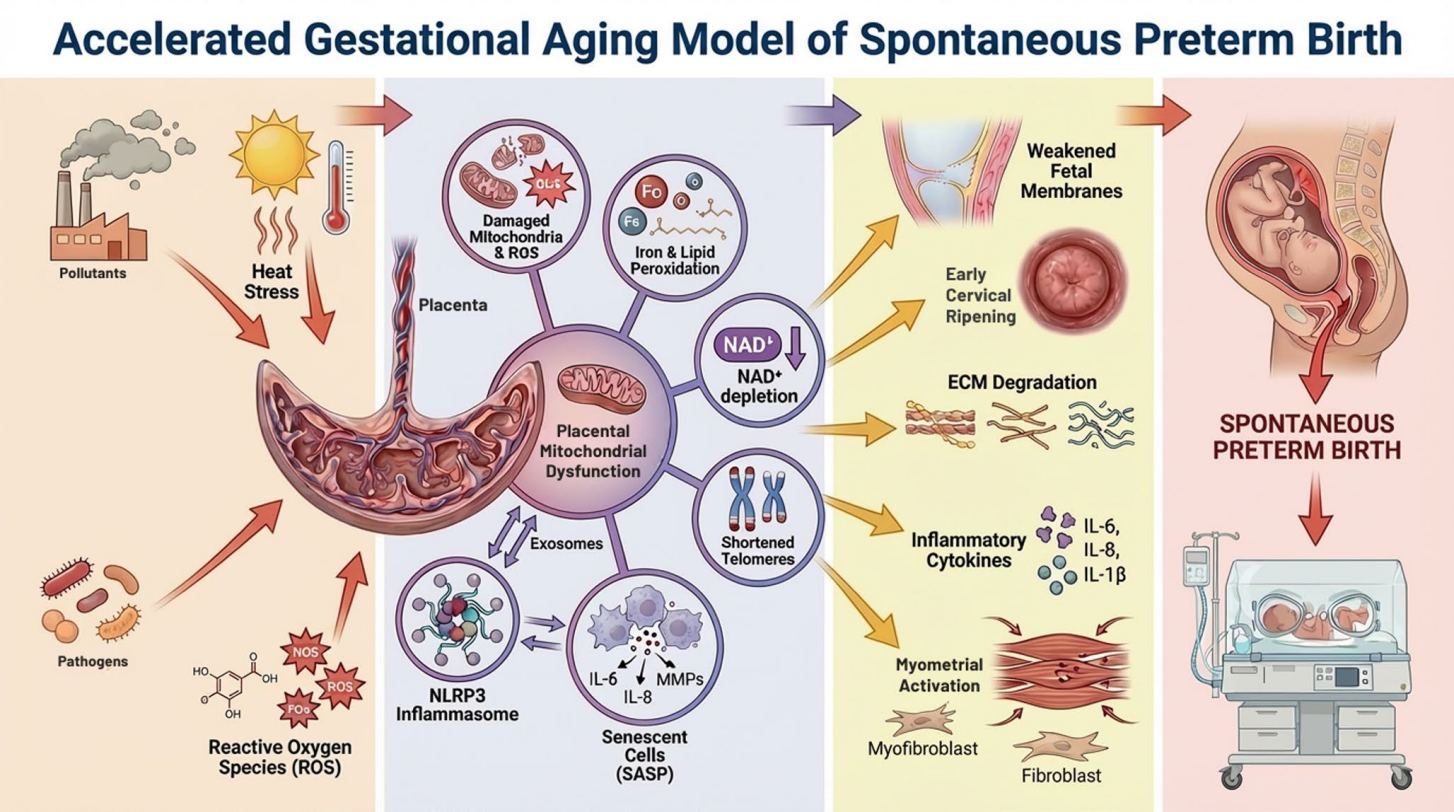
Accelerated gestational aging model of spontaneous preterm birth. Environmental and maternal stressors, including air pollution, heat stress, infection, and oxidative stress, induce mitochondrial dysfunction in gestational tissues. This triggers interconnected pathways such as ferroptosis, NAD⁺ depletion, telomere shortening, cellular senescence with SASP, EV signalling, and NLRP3 inflammasome activation. These processes promote inflammation, extracellular matrix degradation, cervical ripening, fetal membrane weakening, and myometrial activation, culminating in sPTB. ROS, Reactive oxygen species; NAD⁺, Nicotinamide adenine dinucleotide; SASP, Senescence-associated secretory phenotype; NLRP3, NOD-like receptor family pyrin domain containing 3; ECM, Extracellular matrix; sPTB, Spontaneous preterm birth

Normal term parturition involves coordinated molecular changes resembling cellular aging processes through increased oxidative stress, inflammatory signalling, extracellular matrix degradation, and functional senescence of gestational tissues [10]. These changes activate the labour cascade through prostaglandin synthesis, progesterone receptor isoform shifts, cervical ripening, and myometrial contractility [11]. The concept of accelerated gestational or placental aging has been increasingly implicated in adverse pregnancy outcomes, including preterm birth, suggesting that premature activation of aging-related pathways may contribute to sPTB [12]. Central to this model are mitochondrial dysfunction, oxidative stress, and cellular senescence which are hallmarks of aging that have been increasingly implicated in sPTB pathogenesis [11, 13]. This review synthesizes emerging evidence linking these mechanisms to sPTB, with emphasis on underexplored pathways including mitochondrial dynamics, ferroptosis, nicotinamide adenine dinucleotide (NAD^+^) metabolism, inflammasome activation, extracellular vesicle signalling, and deoxyribonucleic acid (DNA) damage responses, while discussing their clinical implications and therapeutic potential. The interconnected molecular mechanisms underlying accelerated gestational aging and premature activation of labour pathways in sPTB are illustrated in Fig. 1.

## Search strategy and selection criteria

This narrative review was conducted using a structured approach to identify relevant literature on sPTB, mitochondrial dysfunction, cellular senescence, and related molecular mechanisms underlying accelerated gestational aging. Relevant literature was identified through searches of major biomedical databases, including PubMed/MEDLINE, along with supplementary screening of reference lists of selected articles and key review papers.

The search strategy incorporated combinations of keywords such as “spontaneous preterm birth (sPTB)”, “preterm labour”, “mitochondrial dysfunction”, “oxidative stress”, “cellular senescence”, “ferroptosis”, “NAD^+^ metabolism”, “telomere shortening”, “inflammasome”, “NLRP3”, “extracellular vesicles”, and “gestational aging”. Emphasis was placed on contemporary literature reflecting advances in molecular, translational, and reproductive biology research, with priority given to studies exploring mechanistic pathways, intercellular signalling, biomarker development, and therapeutic targets in sPTB.

Original research articles, systematic reviews, and high-quality narrative reviews were considered. Studies focusing exclusively on non-human models without translational relevance, non-pregnancy-related conditions, or unrelated biological systems were excluded.

Given the narrative nature of this review, formal risk-of-bias assessment and quantitative synthesis were not performed. Instead, studies were selected based on their relevance, methodological rigor, and contribution to the conceptual understanding of accelerated gestational aging and its role in sPTB pathogenesis. The synthesis was conducted thematically, integrating evidence across mitochondrial biology, oxidative stress, cellular senescence, inflammatory pathways, and extracellular signalling to provide a comprehensive mechanistic framework for sPTB. Having established the conceptual framework of accelerated gestational aging, it is important to recognize that the molecular and cellular processes underlying sPTB occur across interacting maternal and fetal compartments with distinct physiological functions and stress responses. Understanding the tissue-specific biology of these compartments is essential for interpreting how mitochondrial dysfunction, oxidative stress, senescence, inflammation, and extracellular signalling contribute to premature labour.

## Tissue-specific contributions of gestational compartments in sPTB

Although accelerated gestational aging represents a coordinated biological process involving multiple gestational compartments, individual tissues exhibit distinct physiological functions, stress responses, and senescence-associated adaptations that contribute differentially to sPTB.

The placenta primarily functions as a metabolically active organ regulating nutrient transfer, endocrine signalling, oxygen homeostasis, and mitochondrial energy metabolism. Placental dysfunction in sPTB has been associated with mitochondrial oxidative stress, impaired oxidative phosphorylation (OXPHOS), altered metabolic adaptation, and inflammatory signalling, thereby contributing to systemic maternal-fetal stress responses [14, 15].

The fetal membranes, comprising the amnion and chorion, play critical structural, mechanical, and immunological roles in maintaining pregnancy. Increasing evidence suggests that fetal membrane dysfunction represents a major pathological driver of sPTB and PPROM [14]. The amnion is particularly susceptible to oxidative stress-induced senescence because of cumulative exposure to reactive oxygen species (ROS), mechanical stretch, and inflammatory mediators during advancing gestation [16]. Senescent amnion epithelial and fibroblast cells develop a senescence-associated secretory phenotype (SASP), characterized by increased secretion of inflammatory cytokines, matrix metalloproteinases, and damage-associated molecular patterns (DAMPs), which collectively promote extracellular matrix degradation and sterile inflammation [16, 17]. Recent studies further demonstrate that fetal membrane microfractures and structural weakening are associated with cellular senescence and inflammatory remodelling, suggesting that fetal membrane aging is an active biological process contributing to labour initiation rather than passive tissue degeneration [15, 17].

The chorion contributes prominently to immune signalling and inflammatory amplification at the maternal-fetal interface. p38 mitogen-activated protein kinase (MAPK)-mediated senescence has been demonstrated in human chorion trophoblast cells, where oxidative stress-induced activation of senescence pathways promotes inflammatory signalling and cellular aging [18, 19]. Emerging evidence also suggests that heterochromatin erosion, Long Interspersed Nuclear Element-1 (LINE1) derepression, and Serpin Family E Member 1 (SERPINE1)-mediated epithelial senescence within amnion cells may further contribute to propagation of senescence-associated inflammatory signalling during parturition [20, 21].

The decidua functions as a dynamic maternal immune interface regulating immune tolerance, leukocyte recruitment, progesterone responsiveness, and inflammatory signalling preceding labour. Dysregulated maternal-fetal immune homeostasis and intra-amniotic inflammation contribute significantly to sPTB through activation of decidual immune pathways and inflammatory crosstalk between maternal and fetal tissues [22, 23]. The cervix undergoes extensive extracellular matrix remodelling, collagen disorganization, and inflammatory softening during cervical ripening, processes that may be prematurely accelerated under conditions of oxidative stress and inflammation [24].

The myometrium serves as the terminal effector tissue responsible for uterine contractility. Transition of the myometrium from a quiescent to a contractile phenotype involves activation of inflammatory pathways, contraction-associated proteins, gap junction formation, and prostaglandin-mediated signalling cascades [25]. Inflammatory mediators, SASP-associated cytokines, and DAMPs originating from adjacent gestational tissues may collectively contribute to myometrial activation and premature labour initiation. This accelerated gestational aging in sPTB represents a coordinated multicompartment process involving tissue-specific stress responses, senescence pathways, inflammatory signalling, and extracellular matrix remodelling rather than a uniform pathological response across all gestational tissues.

## Mitochondrial dysfunction as a central driver of preterm birth

### Mitochondrial oxidative stress and energy metabolism

Mitochondria serve as cellular powerhouses and critical regulators of oxidative stress, calcium homeostasis, and apoptosis. Placental mitochondrial dysfunction, particularly within trophoblast-rich regions involved in metabolic regulation and endocrine signalling, has emerged as a central feature of sPTB pathogenesis [26]. Recent systematic reviews demonstrate that placental mitochondrial dysfunction, characterized by impaired OXPHOS, reduced adenosine triphosphate (ATP) production, and increased ROS generation, is consistently associated with adverse pregnancy outcomes including sPTB [27]. Transcriptional profiling of preterm placentas reveals downregulation of genes encoding mitochondrial respiratory chain complexes and altered expression of mitochondrial biogenesis regulators such as peroxisome proliferator-activated receptor gamma co-activator 1-α (PGC-1α) and mitochondrial transcription factor A (TFAM) [28]. Human placental studies demonstrate elevated oxidative stress markers, including lipid peroxidation products and protein carbonyls, in fetal membranes from sPTB cases [13]. In contrast, oxidative stress within fetal membrane compartments, particularly the amnion, may contribute more directly to cellular senescence, extracellular matrix degradation, and membrane weakening implicated in PPROM [14, 16].

Maternal exposure to particulate matter with aerodynamic diameter ≤ 2.5 micrometres (PM2.5) exemplifies environmental triggers of mitochondrial dysfunction, with studies showing associations between particulate matter exposure, mitochondrial OXPHOS dysfunction in cord blood, and increased sPTB risk [29]. Heat stress may similarly induce pathophysiological changes at the feto-maternal interface, including mitochondrial dysfunction and metabolic alterations that predispose to preterm birth [3]. These findings underscore the vulnerability of placental mitochondria to environmental stressors and their role in translating external insults into sPTB risk.

### Mitochondrial dynamics, mitophagy, and quality control

Beyond energy production, mitochondrial health depends on dynamic processes of fusion, fission, and selective autophagy of damaged mitochondria, through a process known as mitophagy [30]. Mitochondrial dynamics regulate mitochondrial morphology, distribution, and function, with fusion promoting metabolic efficiency and fission facilitating quality control through mitophagy [31]. Dysregulation of these processes has been implicated in various pathological conditions, yet their specific roles in sPTB remain underexplored.

Recent evidence suggests that impaired mitochondrial quality control contributes to placental dysfunction in sPTB. The regulator of G-protein signalling 12 (RGS12) has emerged as a novel player in placental mitochondrial function [1]. RGS12 localizes to placental mitochondria and regulates ATP-synthase activity through tyrosine phosphorylation of ATP synthase F1 subunit beta (ATP5B). RGS12 deficiency in mice reduces tolerance to preterm birth and decreases fetal weight, while human preterm placentas exhibit reduced RGS12 expression [1]. This suggests that impaired mitochondrial quality control mechanisms, potentially involving defective mitophagy, may contribute to the accumulation of dysfunctional mitochondria in gestational tissues and may promote oxidative stress and inflammatory signalling.

### Mitochondrial DAMPs and sterile inflammation

Damaged mitochondria release DAMPs, including mitochondrial DNA (mtDNA), cardiolipin, and TFAM, which activate innate immune responses [32]. Circulating cell-free mtDNA (ccf-mtDNA) has emerged as a promising biomarker for intra-amniotic infection and inflammation in obstetrics [9]. Elevated mtDNA levels in maternal plasma and amniotic fluid correlate with sPTB risk, particularly in PPROM cases [33]. Mitochondrial DAMPs activate pattern recognition receptors including Toll-like receptor 9 (TLR9) and the NOD-,LRR- and Pyrin Domain-Containing Protein 3 (NLRP3) inflammasome, triggering sterile inflammation even in the absence of microbial infection [34]. This mechanism provides a molecular link between mitochondrial dysfunction and the inflammatory cascade characteristic of sPTB, bridging infection-independent and infection-associated pathways.

Emerging evidence suggests that mitochondrial dysfunction, oxidative stress, and ferroptosis should not be conceptualized as isolated pathogenic mechanisms in sPTB, but rather as interconnected components of a unified redox-metabolic continuum [2, 6, 11, 27, 35]. Mitochondrial dysfunction represents a major upstream source of excessive ROS generation within gestational tissues, promoting lipid peroxidation, mitochondrial membrane injury, DNA damage, and activation of inflammatory signalling pathways [2, 13, 27, 28]. Accumulation of ROS and depletion of antioxidant defences may subsequently facilitate ferroptotic cell death through iron-dependent lipid peroxidation and glutathione peroxidase 4 (GPX4) inactivation [6, 11, 35, 36]. In turn, ferroptotic injury may further exacerbate mitochondrial dysfunction through oxidative damage to mitochondrial membranes and disruption of OXPHOS, thereby creating a self-amplifying cycle of metabolic stress and inflammatory activation [6, 11, 35–37]. This integrated framework links mitochondrial injury, ferroptosis, inflammasome activation, and cellular senescence into a coordinated biological network that contributes to accelerated gestational aging and premature activation of labour pathways in sPTB [5, 34, 38].

## Ferroptosis: an emerging iron-dependent cell death pathway

### Mechanisms of ferroptosis in gestational tissues

Ferroptosis, a recently characterized form of regulated cell death driven by iron-dependent lipid peroxidation, has emerged as a critical mechanism in sPTB pathogenesis [6, 37]. Unlike apoptosis or necrosis, ferroptosis is characterized by accumulation of lipid peroxides, depletion of glutathione, and inactivation of glutathione peroxidase 4 (GPX4), the key enzyme preventing lipid peroxidation [37]. Placental and fetal membrane tissues are particularly vulnerable to ferroptosis due to high metabolic activity, abundant polyunsaturated fatty acids, and iron accumulation during pregnancy [30].

Recent studies suggest that ferroptosis may contribute to trophoblast lipotoxic damage and adverse pregnancy outcomes [37]. Ferroportin, the sole cellular iron exporter, shows dysregulated expression in placentas and fetal membranes from complicated pregnancies, with implications for iron homeostasis and ferroptosis susceptibility [30]. Chorioamnionitis-associated preterm birth exhibits elevated ferroptosis markers, nuclear factor- κB (NF-κB) expression, and antioxidant imbalance, suggesting that ferroptosis contributes to membrane weakening and premature rupture [11]. Furthermore, ferroptosis has been linked to endoplasmic reticulum (ER) stress in preterm birth through targeting of LIM homeobox 1 (LHX1) and Inositol-requiring enzyme 1(IRE-1), revealing crosstalk between ferroptosis and ER stress pathways [26].

### Therapeutic targeting of ferroptosis

The recognition of ferroptosis in sPTB pathogenesis has opened new therapeutic avenues. Ferrostatin-1, a specific ferroptosis inhibitor, demonstrates protective effects against inflammation-induced preterm birth and fetal brain injury in preclinical models [4]. Treatment with ferrostatin-1 reduces lipid peroxidation, preserves membrane integrity, and attenuates inflammatory responses in lipopolysaccharide (LPS)-induced preterm birth models [4]. These findings suggest that ferroptosis inhibitors may represent a potential therapeutic strategy for preventing sPTB, particularly in high-risk pregnancies with evidence of oxidative stress or inflammation.

### Ferroptosis and mitochondrial dysfunction: interconnected pathways

Ferroptosis and mitochondrial dysfunction are intimately connected. Mitochondria are both sources and targets of lipid peroxidation, with mitochondrial ROS promoting ferroptosis and ferroptotic lipid peroxides damaging mitochondrial membranes [37]. This bidirectional relationship creates a vicious cycle wherein mitochondrial dysfunction promotes ferroptosis, and ferroptotic damage further impairs mitochondrial function. Understanding these interconnections is crucial for developing comprehensive therapeutic strategies that address multiple nodes in the oxidative stress-cell death network underlying sPTB.

## Nicotinamide adenine dinucleotide (NAD^+^) metabolism and sirtuin signalling in gestational aging

### NAD^+^ depletion and metabolic dysfunction

NAD^+^ is a critical cofactor for cellular energy metabolism and a substrate for NAD^+^-consuming enzymes including sirtuins, poly (ADP-ribose) polymerases (PARPs), and cluster of differentiation 38 (CD38) [39]. NAD^+^ levels decline with aging and oxidative stress, contributing to metabolic dysfunction and cellular senescence [40]. Although direct evidence linking NAD^+^ metabolism to sPTB remains limited, several lines of evidence suggest its involvement. Placental mitochondrial dysfunction and reduced OXPHOS capacity in sPTB likely involve NAD^+^ depletion, as NAD^+^ is essential for mitochondrial respiratory chain function [27, 28].

### Sirtuins as guardians of gestational health

Sirtuins, NAD^+^-dependent deacetylases, regulate diverse cellular processes including mitochondrial biogenesis, oxidative stress responses, inflammation, and cellular senescence [41]. Sirtuin 1 (SIRT1), the most extensively studied sirtuin, promotes mitochondrial function through deacetylation of PGC-1α, enhances antioxidant defences through activation of forkhead box O (FOXO) transcription factors, and suppresses inflammatory signalling through NF-κB inhibition [41]. Sirtuin 3 (SIRT3), localized to mitochondria, regulates mitochondrial metabolism and protects against oxidative stress [41]. Reduced sirtuin activity in gestational tissues could contribute to mitochondrial dysfunction, oxidative stress, and premature senescence characteristic of sPTB.

### Therapeutic potential of NAD^+^ boosting strategies

NAD^+^ precursors including nicotinamide riboside (NR) and nicotinamide mononucleotide (NMN) have shown promise in preclinical aging models, improving mitochondrial function, reducing oxidative stress, and extending cellular health [40]. While clinical data in pregnancy are lacking, the potential of NAD^+^ boosting strategies to enhance placental mitochondrial function and delay gestational aging warrants investigation. Such interventions could complement antioxidant therapies by addressing upstream metabolic dysfunction rather than merely scavenging ROS.

## Telomere biology and DNA damage responses in preterm birth

### Telomere shortening as a marker of cellular aging

Telomeres, protective nucleoprotein structures at chromosome ends, shorten with each cell division and serve as molecular clocks of cellular aging [42]. Critically short telomeres trigger DNA damage responses, cell cycle arrest, and cellular senescence [43]. Telomere shortening within fetal membrane compartments, particularly the amnion and chorion, has been consistently associated with sPTB and PPROM, supporting the concept that premature fetal membrane senescence contributes to inflammatory activation, extracellular matrix remodelling, and membrane destabilization [7, 14–16, 44]. Studies demonstrate that fetal membranes from PPROM pregnancies exhibit significantly shorter telomeres compared to term controls, with telomere length inversely correlating with gestational age at delivery [7, 32].

Placental telomere dynamics during pregnancy reveal intriguing patterns. While some studies report stable telomere length throughout gestation, recent evidence shows an increase in short telomeres during the third trimester in human placenta, suggesting accelerated cellular turnover or senescence as term approaches [8]. This physiological telomere shortening may represent a normal aging process that, when accelerated, contributes to sPTB [5]. Maternal peripheral blood telomere length has also been associated with sPTB risk in some populations, though findings remain inconsistent across studies [39, 40].

### DNA damage, oxidative stress, and telomere attrition

Oxidative stress accelerates telomere shortening due to the high guanine content of telomeric DNA, which is particularly vulnerable to oxidative damage [5]. Environmental exposures including air pollution demonstrate complex relationships with telomere length and sPTB risk. A U-shaped relationship between ozone exposure and preterm birth risk has been reported, with associations modified by preconception telomere length [31]. This suggests that telomere length may modulate individual susceptibility to environmental stressors, with shorter telomeres conferring reduced resilience to oxidative insults.

### Telomere fragments as DAMPs

Beyond serving as markers of cellular aging, telomere fragments released from senescent cells act as DAMPs, activating inflammatory responses. Telomere fragments in amniotic fluid have been detected in association with fetal membrane senescence and sPTB. These fragments, along with high-mobility group box 1 (HMGB1), contribute to sterile inflammation and may propagate senescence to neighbouring cells through paracrine signalling [38]. This mechanism links telomere dysfunction to the inflammatory cascade characteristic of sPTB, providing another pathway through which cellular aging drives premature labour.

## Cellular senescence and the SASP

### Senescence pathways in gestational tissues

Cellular senescence, a state of stable cell cycle arrest accompanied by altered gene expression and secretory activity, represents a fundamental aging mechanism [43]. Senescent cells accumulate across multiple gestational compartments as pregnancy progresses, although the biological consequences differ between tissues. In fetal membranes, particularly the amnion, senescence promotes sterile inflammation, extracellular matrix remodelling, and membrane weakening, whereas in decidual tissues senescence is more closely linked to immune activation and inflammatory signalling preceding labour [14, 16, 22, 45]. Multiple stressors can induce senescence in gestational tissues, including oxidative stress, telomere dysfunction, DNA damage, and inflammatory signals [46]. The p53/p21 and p16INK4a/retinoblastoma (Rb) pathways represent the two major senescence-inducing pathways, with evidence for activation of both in sPTB [41].

Long non-coding ribonucleic acids (lncRNAs) have emerged as regulators of cellular senescence in sPTB. Small Nucleolar RNA Host Gene 29 (SNHG29), a lncRNA, regulates cell senescence via p53/p21 signalling in sPTB, with altered SNHG29 expression in fetal membranes from sPTB cases [41]. This highlights the complexity of senescence regulation and identifies novel molecular targets for therapeutic intervention.

### The SASP

Senescent cells develop a SASP, characterized by secretion of pro-inflammatory cytokines [interleukin (IL)-6, IL-8, IL-1β], chemokines, growth factors, and matrix metalloproteinases (MMPs) [47]. The SASP has paradoxical effects while being beneficial for wound healing and tumour suppression in some contexts, chronic SASP contributes to tissue dysfunction and age-related pathologies [47]. In gestational tissues, the SASP promotes inflammatory signalling, extracellular matrix degradation, and paracrine senescence, creating a pro-labour environment [45]. Recent evidence further suggests that senescence-associated inflammatory signalling differs substantially between gestational compartments. Amnion epithelial and fibroblast cells exhibit increased release of SASP-associated cytokines, DAMPs, extracellular vesicles, and MMPs capable of propagating paracrine senescence and inflammatory amplification across fetal membranes [16, 17]. In contrast, chorionic trophoblast cells demonstrate prominent p38 MAPK-mediated senescence responses linked to inflammatory activation and oxidative stress adaptation [18, 19].

Galectin-3, a β-galactoside-binding lectin, has been identified as a marker of inflammation and senescence in sPTB [32]. Elevated galectin-3 levels and telomere shortening in fetal membranes from sPTB cases suggest a common pathway of enhanced inflammation and senescence [32]. Galectin-3 promotes inflammatory responses and may contribute to membrane weakening and premature rupture.

### p38 MAPK and cyclic GMP-AMP synthase-stimulator of interferon genes (cGAS-STING) pathways linking DNA damage to senescence

The progression from localized cellular injury to the sterile inflammatory state in sPTB is driven by interconnected stress-response and innate immune pathways, particularly the p38 MAPK pathway, the cGAS-STING axis.

The p38 MAPK pathway functions as a highly conserved, stress-responsive intracellular sensor that regulates cell cycle arrest and drives premature cellular senescence in the fetal membranes [18, 19]. Concurrently, the cGAS-STING pathway serves as an important innate immune surveillance mechanism designed to detect misplaced cytosolic double-stranded DNA. Upon interacting with cytosolic DNA, cGAS catalyses the synthesis of cyclic GMP-AMP, which subsequently binds STING to propagate a robust pro-inflammatory and type I interferon response [20]. These localised signalling events are key drivers that may ultimately culminate in the hyperactive secretory state of the SASP.

Oxidative stress, progressive telomere attrition, and environmental toxicants together induce profound genomic instability within the fetal membranes, leading to the accumulation of damaged DNA and the persistent activation of DNA damage response pathways [5, 16, 32, 48]. Within this altered microenvironment, the p38 MAPK pathway emerges as a central mechanistic regulator. Continuous oxidative stress activates p38 MAPK signalling, which promotes the development and amplification of the SASP [16, 48]. Recent primary research utilizing Clustered Regularly Interspaced Short Palindromic Repeats (CRISPR)/CRISPR-associated protein 9 (Cas9) knockout models demonstrated that silencing p38 MAPK in human fetal chorion trophoblast cells completely prevented oxidative stress-induced senescence and significantly reduced the release of pro-inflammatory SASP factors, confirming its master regulatory role in translating oxidative damage into inflammatory cascades [18]. Furthermore, inhibiting the p38 MAPK pathway using statins has been shown to effectively downregulate oxidative stress-induced senescence and SASP in fetal membranes, highlighting a promising avenue to uncouple DNA damage from premature labour initiation [19].

In parallel, the cGAS-STING pathway has recently emerged as an important innate immune sensor within gestational tissues, acting as a molecular link between sublethal cellular injury and sterile inflammation. In human amnion fibroblasts, nuclear translocation of the protein S100A9 causes heterochromatin erosion, which consequently de-represses LINE1 at parturition. The subsequent retro transposition of LINE1 leads to the accumulation of cytosolic cDNA, which is recognized by cGAS. Experimental evidence suggests that activation of the cGAS-STING pathway triggers type I interferon responses and may promote cellular senescence with an amplified SASP and increased prostaglandin E2 production [20].

Crucially, these localized senescence mechanisms are actively propagated to adjacent healthy cells through intricate paracrine signalling. For instance, increased expression of SERPINE1 by amnion fibroblasts can induce senescence in adjacent amnion epithelial cells by triggering the shedding of vitronectin and interrupting cell adhesion pathways [21]. Similarly, extracellular vesicles derived from amniotic epithelial cells further coordinate this widespread cellular transition [16, 48]. The synergistic amplification of the p38 MAPK and cGAS-STING pathways, reinforced by paracrine signalling loops, perfectly illustrates how localized DNA damage and oxidative stress are translated into the widespread, irreversible inflammatory activation observed in sPTB [18, 20, 21]. Targeting these specific mechanistic pathways may therefore provide novel precision-based therapeutic strategies to disrupt the link between DNA damage and premature initiation of labour.

### Senolytic therapies: targeting senescent cells

The recognition that senescent cells contribute to sPTB has generated interest in senolytic therapies, agents that selectively eliminate senescent cells. Senolytics including dasatinib plus quercetin, navitoclax, and fisetin have shown efficacy in preclinical aging models, improving tissue function and reducing age-related cellular dysfunction [49]. While clinical data in pregnancy are lacking, proof-of-concept studies in animal models could establish whether senolytic interventions delay gestational aging and prevent sPTB. Such approaches would require careful safety evaluation given the potential effects on fetal development.

## Inflammasome activation and pyroptosis in preterm labour

### The NLRP3 inflammasome in intra-amniotic inflammation

The NLRP3 inflammasome, a multiprotein complex that activates caspase-1 and promotes IL-1β and IL-18 secretion, plays a central role in innate immunity and sterile inflammation. Intra-amniotic inflammation, whether infection-associated or sterile, activates the NLRP3 inflammasome in fetal membranes and decidua, triggering preterm labour. LPS-induced intra-amniotic inflammation in animal models demonstrates NLRP3 inflammasome priming and canonical activation, with increased expression of NLRP3, caspase-1, and IL-1β in gestational tissues [34]. Decidual immune cells and leukocyte trafficking at the maternal-fetal interface further amplify inflammasome-associated inflammatory signalling and contribute to propagation of intra-amniotic inflammation preceding preterm labour [22, 23].

Critically, pharmacological inhibition of NLRP3 with methyl-2-(2-chlorophenyl)-2-(2,5-dimethoxyphenyl) acetate called as MCC950 reduces intra-amniotic inflammation-induced preterm birth and neonatal mortality in preclinical models [34]. These findings provide strong experimental evidence supporting a mechanistic role for NLRP3 inflammasome activation in inflammation-induced preterm birth and identify the inflammasome as a potential therapeutic target. IL-37, an anti-inflammatory cytokine, alleviates inflammatory effects and NLRP3 inflammasome activation in LPS-induced preterm birth through suppression of the NF-κB/NLRP3 axis [42], suggesting that endogenous anti-inflammatory mechanisms may be harnessed therapeutically.

### Inflammasome activation by mitochondrial DAMPs

Mitochondrial DAMPs, including mtDNA and cardiolipin, are potent activators of the NLRP3 inflammasome. Mitochondrial dysfunction and oxidative stress promote mitochondrial membrane permeabilization and release of these DAMPs into the cytosol, where they trigger inflammasome assembly [34]. This mechanism links mitochondrial dysfunction to inflammatory activation in sPTB, providing a molecular pathway through which oxidative stress translates into inflammatory signalling. The convergence of mitochondrial dysfunction, DAMP release, and inflammasome activation represents a critical node in sPTB pathogenesis and a potential therapeutic target.

### Pyroptosis and inflammatory cell death

**Fig. 2:**
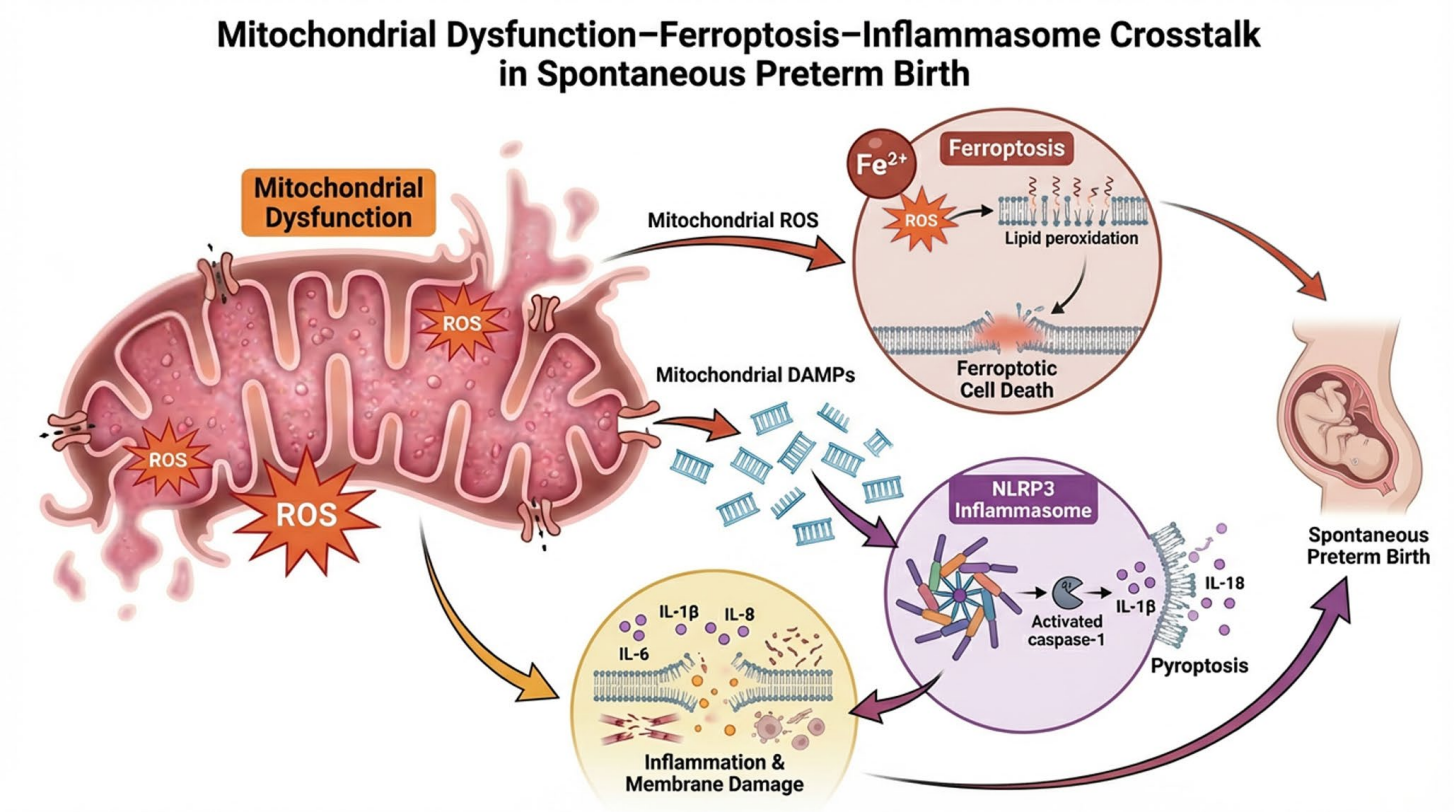
Mitochondrial dysfunction-ferroptosis-inflammasome crosstalk in spontaneous preterm birth. Mitochondrial dysfunction increases mitochondrial ROS, promoting iron-dependent lipid peroxidation and ferroptotic cell death. Damaged mitochondria release mitochondrial DAMPs, including mitochondrial DNA fragments, which activate the NLRP3 inflammasome and caspase-1-mediated pyroptosis. These interconnected pathways amplify inflammatory signalling and membrane injury, contributing to sPTB. Therapeutic interventions targeting key nodes within this network have demonstrated beneficial effects in preclinical models, including Ferrostatin-1, which inhibits ferroptosis, and MCC950, which suppresses NLRP3 inflammasome activation. ROS, Reactive oxygen species; DAMPs, Damage-associated molecular patterns; NLRP3, NOD-like receptor family pyrin domain containing 3; sPTB, Spontaneous preterm birth

Inflammasome activation leads to pyroptosis, a form of inflammatory cell death characterized by cell swelling, membrane rupture, and release of inflammatory cytokines. Pyroptosis in gestational tissues contributes to membrane weakening, inflammatory amplification, and labour activation. Unlike apoptosis, which is immunologically silent, pyroptosis actively promotes inflammation through release of intracellular contents including additional DAMPs [50]. Targeting pyroptosis, either through inflammasome inhibition or caspase-1 blockade, represents a strategy for limiting inflammatory tissue damage in sPTB. These interconnected pathways demonstrate how mitochondrial dysfunction, ferroptosis, and inflammasome activation collectively amplify inflammatory signalling and cellular injury in gestational tissues, ultimately contributing to premature labour activation. The mechanistic crosstalk between these pathways is summarized in Fig. 2.

## Extracellular vesicles as mediators of intercellular communication

### Exosome biogenesis and cargo

Extracellular vesicles (EVs), including exosomes (30–150 nm) and micro vesicles (100–1000 nm), mediate intercellular communication by transferring proteins, lipids, and nucleic acids between cells [51]. Placental syncytiotrophoblasts release abundant EVs into maternal circulation, with EV concentrations increasing throughout pregnancy [52]. EV cargo reflects the physiological or pathological state of parent cells, with altered EV composition in pregnancy complications including sPTB [53].

Placenta-specific microRNAs packaged in exosomes regulate maternal immune responses and vascular function [54]. Syncytiotrophoblasts-derived EVs modulate maternal immune tolerance and inflammatory responses, with dysregulated EV signalling implicated in pregnancy complications [52]. The dynamic changes in EV composition and concentration throughout pregnancy suggest that EVs participate in the physiological timing of parturition, with aberrant EV signalling contributing to sPTB [53].

### Senescence-associated EVs and inflammatory signalling

Senescent cells release EVs with distinct cargo, including SASP factors, microRNAs, and DAMPs [53]. Senescence-associated EVs from amnion cells contain HMGB1 and telomere fragments, which activate inflammatory responses in recipient cells and propagate senescence [38, 53]. In preclinical models, exosomes from senescent amnion cells induced preterm birth in mice, suggesting that senescence-associated EVs may contribute to establishing a causal role for senescence-associated EVs in sPTB [53]. This mechanism provides a pathway for systemic dissemination of senescence signals across gestational tissues, coordinating premature labour activation. Within fetal membranes, senescent amnion cells release EVs enriched with inflammatory mediators and DAMPs capable of propagating senescence-associated signalling to adjacent cells, thereby contributing to coordinated inflammatory activation across gestational compartments [17].

### EV biomarkers for preterm birth prediction

The accessibility of circulating EVs in maternal blood makes them attractive biomarker candidates for sPTB prediction. Alpha-2-macroglobulin (A2M) in circulating exosome-like vesicles is significantly elevated in women with preterm pregnancies, suggesting A2M-containing EVs as potential biomarkers for sPTB risk [43]. EV-associated proteins and microRNAs reflecting placental stress, inflammation, or senescence could enable early identification of high-risk pregnancies, facilitating targeted interventions. Multi-marker panels combining EV biomarkers with clinical risk factors may improve predictive accuracy beyond current approaches.

## Oxidative stress and antioxidant defence systems

### Sources of ROS in pregnancy

ROS are generated through multiple interconnected pathways across gestational tissues, with dysfunctional placental and fetal membrane mitochondria representing major upstream sources of pathological oxidative stress [2, 13, 27, 28]. While physiological ROS levels participate in normal cellular signalling, excessive ROS accumulation within trophoblasts, amnion epithelial cells, chorion trophoblasts, and decidual tissues overwhelms antioxidant defence systems and promotes oxidative damage to lipids, proteins, and DNA [5, 14, 16, 55]. Importantly, mitochondrial ROS also function as critical upstream mediators of ferroptotic signalling by driving iron-dependent lipid peroxidation and membrane injury [6, 11, 35, 36]. The resulting lipid peroxides further impair mitochondrial OXPHOS and membrane integrity, thereby establishing a self-amplifying cycle linking mitochondrial dysfunction, ferroptosis, sterile inflammation, inflammasome activation, and cellular senescence across gestational compartments [5, 11, 34–36]. Environmental exposures including air pollution, heat stress, and toxins, together with maternal metabolic conditions such as obesity and diabetes, further amplify these interconnected redox-inflammatory pathways in pregnancy [3, 10, 29, 33].

### Antioxidant defence mechanisms and their dysregulation

Cells employ multiple antioxidant defence systems, including enzymatic antioxidants (superoxide dismutase, catalase, glutathione peroxidase) and non-enzymatic antioxidants (glutathione, vitamins C and E) [55]. The nuclear factor erythroid 2-related factor 2- Kelch-like ECH-associated protein 1- Antioxidant response element (Nrf2-Keap1-ARE) pathway represents a master regulator of antioxidant responses, with Nrf2 activation inducing expression of antioxidant and detoxification enzymes. Preterm placentas exhibit reduced Nrf2 activity and antioxidant enzyme expression, contributing to oxidative stress accumulation [2].

Targeting mitochondrial oxidative stress with MitoTEMPO, a mitochondria-targeted antioxidant, protects against preterm birth and fetal brain injury in preclinical models. MitoTEMPO ameliorates maternal oxidative stress and inflammation, particularly in placentas, through Nrf2 pathway activation [2]. This demonstrates that mitochondria-targeted antioxidants may be more effective than general antioxidants for preventing sPTB, as they address oxidative stress at its primary source.

### Antioxidant imbalance in chorioamnionitis

Chorioamnionitis-associated preterm birth exhibits profound antioxidant imbalance, with elevated oxidative stress markers and reduced antioxidant capacity [11]. This imbalance contributes to membrane weakening through lipid peroxidation, protein oxidation, and activation of MMPs [17]. The combination of oxidative stress, ferroptosis, and NF-κB activation in chorioamnionitis creates a self-amplifying cycle of inflammation and tissue damage [11]. Therapeutic strategies addressing antioxidant imbalance, potentially through Nrf2 activators or mitochondria-targeted antioxidants, may prevent progression from subclinical inflammation to overt preterm labour.

Collectively, these interconnected pathways including mitochondrial dysfunction, ferroptosis, NAD⁺ metabolism, telomere attrition, cellular senescence, inflammasome activation, EV signalling, and oxidative stress contribute to accelerated gestational aging and premature activation of labour pathways. A summary of the major molecular mechanisms implicated in sPTB are presented in Table 1.

**Table 1:** Major molecular mechanisms driving accelerated gestational aging in spontaneous preterm birth

Mechanistic pathway	Key molecular events	Evidence in sPTB	Potential therapeutic targets
Mitochondrial dysfunction	Impaired OXPHOS, ATP depletion, increased ROS generation	Placental mitochondrial damage and increased oxidative stress markers in preterm membranes	MitoTEMPO, MitoQ, mitochondrial antioxidants
Ferroptosis	Iron-dependent lipid peroxidation, GPX4 inactivation	Increased ferroptosis markers in chorioamnionitis-associated sPTB	Ferrostatin-1, iron homeostasis modulation
NAD⁺ metabolism and sirtuins	Reduced NAD⁺ levels, decreased SIRT1/SIRT3 activity	Associated with mitochondrial dysfunction and oxidative stress in gestational tissues	Nicotinamide riboside, NMN
Telomere attrition and DNA damage	Telomere shortening, activation of DDR pathways	Shortened telomeres in fetal membranes and placenta in PPROM	Antioxidant therapies, telomere-stabilizing strategies
Cellular senescence and SASP	p53/p21 and p16 activation, secretion of IL-6, IL-8, MMPs	Accelerated senescence observed in fetal membranes and placenta	Senolytics, SASP modulators
Inflammasome activation	NLRP3 activation, caspase-1 cleavage, IL-1β release	Increased inflammasome signalling in intra-amniotic inflammation	MCC950, IL-37
Extracellular vesicle signalling	Transfer of inflammatory proteins, microRNAs, and DAMPs	Senescence-associated EVs induce preterm birth in animal models	EV-based biomarkers, signalling modulation

This table summarizes the principal molecular mechanisms implicated in sPTB within the framework of accelerated gestational aging. These interconnected pathways including mitochondrial dysfunction, ferroptosis, telomere attrition, cellular senescence, inflammasome activation, and EV signalling collectively promote oxidative stress, inflammatory responses, and tissue remodelling across gestational compartments. The table also highlights emerging therapeutic targets aimed at modulating these pathogenic pathways

ATP: Adenosine triphosphate; DAMPs: Damage-associated molecular patterns; DDR: DNA damage response; EV: Extracellular vesicle; GPX4: Glutathione peroxidase 4; IL: Interleukin; MCC950: NLRP3 inflammasome inhibitor; MMPs: Matrix metalloproteinases NAD⁺: Nicotinamide adenine dinucleotide; NLRP3: NOD-like receptor family pyrin domain containing 3; NMN: Nicotinamide mononucleotide; OXPHOS: Oxidative phosphorylation; PPROM: Preterm premature rupture of membranes; ROS: Reactive oxygen species; SASP: Senescence-associated secretory phenotype; SIRT: Sirtuin; sPTB: Spontaneous preterm birth

## Clinical biomarkers and predictive models

### Circulating mitochondrial DNA as a biomarker

ccf-mtDNA has emerged as a promising biomarker for intra-amniotic infection and inflammation, with elevated levels reflecting mitochondrial dysfunction and acting as a DAMP that activates innate immune pathways. Elevated ccf-mtDNA has also been linked to inflammatory activation and adverse pregnancy outcomes, including preterm birth [9]. The development of rapid, point-of-care assays for ccf-mtDNA could enable real-time risk assessment and guide clinical decision-making regarding interventions such as corticosteroids or tocolysis.

### Multi-marker panels integrating mechanistic pathways

Single biomarkers rarely achieve sufficient predictive accuracy for clinical implementation. Multi-marker panels integrating biomarkers reflecting different mechanistic pathways like mitochondrial dysfunction (ccf-mtDNA), oxidative stress (lipid peroxidation products), senescence (galectin-3, p16INK4a), inflammation (IL-6, IL-8), and EV cargo (A2M, specific microRNAs) may improve sPTB prediction [32, 43]. Proteomic and metabolomic approaches have identified temporal changes in maternal plasma proteins and metabolites throughout pregnancy, with distinct signatures preceding preterm birth [56]. Machine learning algorithms integrating clinical risk factors with molecular biomarkers could enable personalized risk stratification and targeted interventions.

Despite the growing interest in biomarker discovery for sPTB, several important translational challenges currently limit clinical implementation. Many candidate biomarkers demonstrate substantial gestational age-dependent variability, and the optimal timing of sample collection for accurate risk stratification remains incompletely defined [57, 58]. Furthermore, circulating biomarkers such as ccf-mtDNA, inflammatory cytokines, oxidative stress markers, and EV cargo may not reliably distinguish tissue-specific pathological processes occurring within the placenta, fetal membranes, decidua, cervix, or myometrium [57, 59]. The biological heterogeneity of sPTB further complicates biomarker interpretation, as distinct pathogenic pathways may predominate across different clinical phenotypes [57]. In addition, methodological variability in sample handling, EV isolation techniques, assay platforms, and biomarker quantification contributes to inconsistent reproducibility across studies and currently limits standardization for routine clinical use [58]. Consequently, although multi-marker strategies remain promising, large prospective longitudinal studies incorporating standardized protocols, temporal profiling across gestation, and external validation across diverse populations will be essential before these biomarkers can be reliably translated into precision-based clinical practice.

### Telomere length and environmental susceptibility

Maternal telomere length may serve as a marker of biological aging and susceptibility to environmental stressors [39, 40]. While associations between maternal telomere length and sPTB risk vary across populations, telomere length may identify women at increased risk when exposed to environmental toxins or oxidative stressors [31]. Integrating telomere length with environmental exposure data could refine risk prediction and guide preventive strategies including antioxidant supplementation or exposure reduction.

Taken together, these findings highlight the potential utility of integrating biomarkers reflecting mitochondrial dysfunction, oxidative stress, cellular senescence, and EV signalling for early prediction of sPTB. Key candidate biomarkers and their clinical relevance are summarized in Table 2.

**Table 2:** Emerging molecular biomarkers for prediction of spontaneous preterm birth

Biomarker	Biological pathway reflected	Sample source	Clinical relevance
Cell-free mitochondrial DNA	Mitochondrial dysfunction and DAMP signalling	Maternal plasma, amniotic fluid	Associated with intra-amniotic inflammation and increased risk of PPROM
Telomere length	Cellular aging and oxidative stress	Maternal blood, placental tissue	May reflect biological aging and susceptibility to environmental stressors
Galectin-3	Senescence-associated inflammation	Fetal membranes, maternal circulation	Marker of inflammatory signalling and tissue senescence
Oxidative stress biomarkers	Lipid peroxidation and ROS-mediated damage	Maternal urine or plasma	Associated with increased risk of preterm birth
Extracellular vesicle cargo (A2M, microRNAs)	Intercellular signalling and placental stress responses	Maternal circulation	Potential early biomarkers reflecting placental dysfunction
Proteomic pregnancy clock markers	Temporal molecular signatures of gestational aging	Maternal plasma	May enable early identification of pregnancies at risk for sPTB

This table summarizes emerging biomarkers reflecting key biological pathways involved in sPTB, including mitochondrial dysfunction, oxidative stress, cellular senescence, and EV signalling. Integration of these molecular biomarkers with clinical risk factors may improve early prediction of sPTB and facilitate personalized risk stratification and targeted preventive interventions

A2M: Alpha-2-macroglobulin; DAMPs: Damage-associated molecular patterns; EV: Extracellular vesicle; PPROM: Preterm premature rupture of membranes; ROS: Reactive oxygen species; sPTB: Spontaneous preterm birth

## Therapeutic strategies and future directions

### Mitochondria-targeted interventions

Mitochondria-targeted antioxidants including MitoQ and MitoTEMPO represent potential therapeutic strategies for sPTB prevention [2]. These compounds accumulate in mitochondria through conjugation to lipophilic cations, achieving high local concentrations at the site of ROS generation [2]. Preclinical studies demonstrate that MitoTEMPO reduces inflammation-induced preterm birth and fetal brain injury through Nrf2 pathway activation [2]. Clinical translation will require safety studies in pregnancy, dose optimization, and identification of patient populations most likely to benefit. Emerging insights into the molecular pathways driving accelerated gestational aging have also facilitated the identification of potential biomarkers and targeted therapeutic strategies for sPTB.

**Fig. 3:**
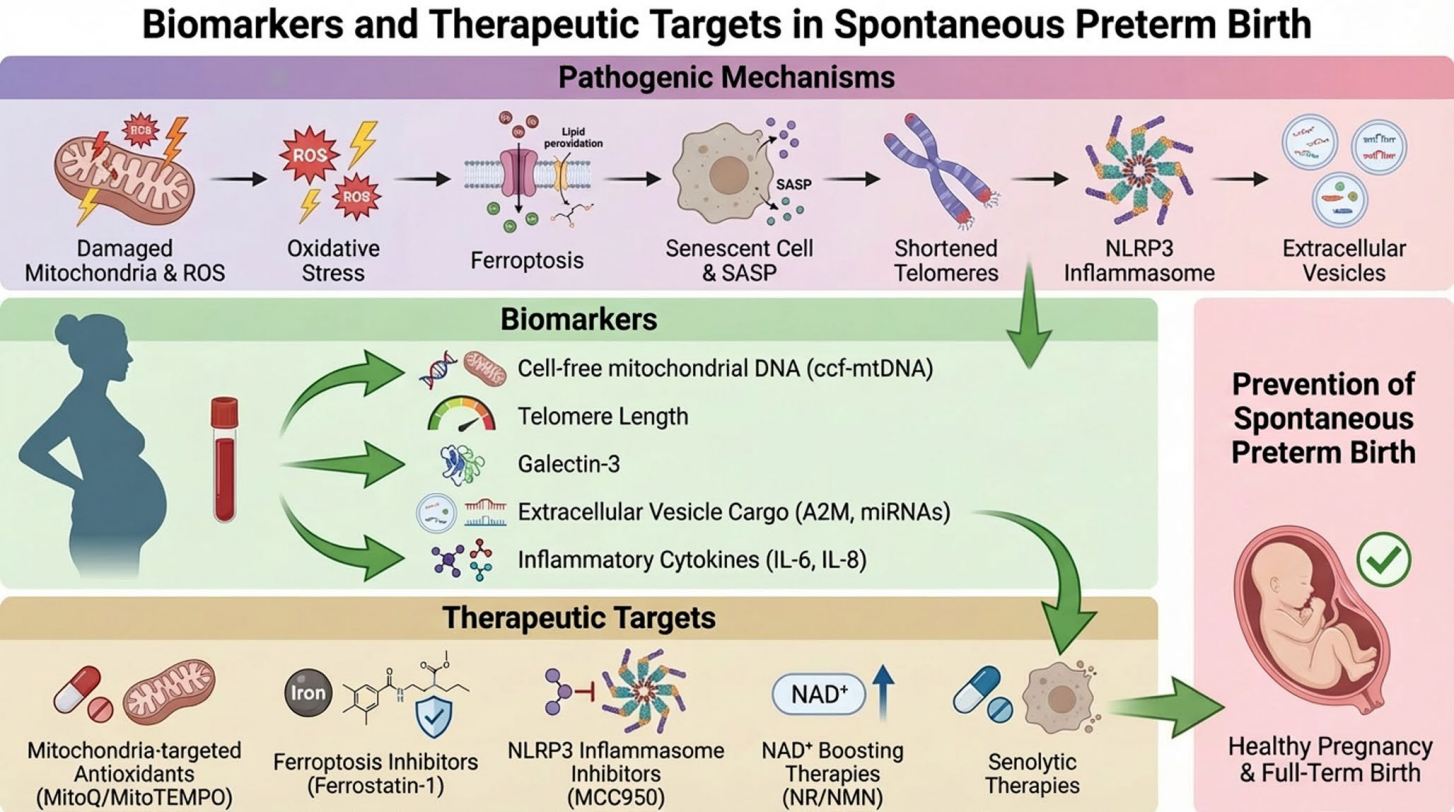
Biomarkers and therapeutic targets in spontaneous preterm birth. Key pathogenic pathways including mitochondrial dysfunction, oxidative stress, ferroptosis, cellular senescence, telomere shortening, NLRP3 inflammasome activation, and EV signalling generate measurable biomarkers in maternal circulation. Candidate biomarkers include ccf-mtDNA, telomere length, galectin-3, EV cargo, and inflammatory cytokines. These mechanisms also provide therapeutic targets such as mitochondria-targeted antioxidants (MitoQ, MitoTEMPO), ferroptosis inhibitors (ferrostatin-1), NLRP3 inhibitors (MCC950), NAD⁺-boosting therapies (NR, NMN), and senolytic agents. ccf-mtDNA, circulating cell-free mitochondrial DNA; NAD⁺, Nicotinamide adenine dinucleotide; NR, Nicotinamide riboside; NMN, Nicotinamide mononucleotide

NAD^+^ precursors including NR and NMN offer another approach to enhancing mitochondrial function. By boosting NAD^+^ levels, these compounds could improve mitochondrial respiration, enhance sirtuin activity, and reduce oxidative stress [40]. While clinical data in pregnancy are lacking, the safety profile of nicotinamide derivatives and their potential to address upstream metabolic dysfunction warrant investigation. A translational overview linking pathogenic mechanisms with biomarker discovery and therapeutic interventions is illustrated in Fig. 3.

### Inflammasome inhibitors and anti-inflammatory strategies

NLRP3 inflammasome inhibitors including MCC950 demonstrate efficacy in preclinical models of inflammation-induced preterm birth. These agents selectively block NLRP3 activation without broadly suppressing immunity, potentially offering advantages over non-specific anti-inflammatory drugs [34]. IL-37, an endogenous anti-inflammatory cytokine, represents another therapeutic approach, with recombinant IL-37 reducing inflammasome activation and preterm birth in animal models [42]. Clinical development of inflammasome-targeted therapies for sPTB prevention requires careful consideration of timing, as some inflammatory processes are necessary for normal labour initiation at term.

### Ferroptosis inhibitors and iron homeostasis

Ferrostatin-1 and other ferroptosis inhibitors protect against inflammation-induced preterm birth and fetal brain injury in preclinical models as discussed earlier. These compounds prevent lipid peroxidation and preserve membrane integrity, addressing a key mechanism of membrane weakening in PPROM [4]. Clinical translation will require development of compounds with favourable pharmacokinetics and safety profiles in pregnancy. Strategies to optimize iron homeostasis, including modulation of ferroportin expression, may complement ferroptosis inhibitors [30].

### Senolytic therapies and cellular rejuvenation

Senolytic agents that selectively eliminate senescent cells represent a novel therapeutic approach. While clinical data in pregnancy are lacking, proof-of-concept studies in animal models could establish whether senolytic interventions delay gestational aging and prevent sPTB [49]. The timing of senolytic administration would be critical, as some degree of senescence may be necessary for normal labour initiation at term. Alternatively, strategies to modulate the SASP without eliminating senescent cells, such as Janus kinase (JAK) inhibitors or mechanistic target of rapamycin (mTOR) inhibitors, may offer a more nuanced approach [60].

Emerging therapeutic strategies targeting mitochondrial dysfunction, ferroptosis, inflammasome activation, and cellular senescence may offer promising avenues for preventing premature activation of labour pathways. A summary of these potential therapeutic approaches and their mechanisms of action is provided in Table 3.

**Table 3:** Emerging therapeutic strategies targeting accelerated gestational aging in spontaneous preterm birth

Therapeutic strategy	Targeted biological pathway	Mechanism of action	Evidence and potential clinical relevance
Mitochondria-targeted antioxidants (MitoTEMPO, MitoQ)	Mitochondrial oxidative stress	Selectively scavenge mitochondrial reactive oxygen species and restore mitochondrial function	Preclinical studies demonstrate protection against inflammation-induced preterm birth and fetal injury
NAD⁺ precursors (nicotinamide riboside, nicotinamide mononucleotide)	Metabolic dysfunction and mitochondrial aging	Enhance NAD⁺ availability, improve mitochondrial respiration, and activate sirtuin signalling	Shown to improve mitochondrial function in aging models; potential for improving placental metabolism
Ferroptosis inhibitors (Ferrostatin-1)	Iron-dependent lipid peroxidation	Prevent lipid peroxidation and preserve cellular membrane integrity	Demonstrated protective effects against inflammation-induced preterm birth in experimental models
Inflammasome inhibitors (MCC950)	NLRP3 inflammasome activation	Block inflammasome assembly and reduce IL-1β-mediated inflammatory signalling	Reduces intra-amniotic inflammation and preterm birth in animal models
Anti-inflammatory cytokine therapy (IL-37)	NF-κB-mediated inflammatory signalling	Suppresses inflammatory pathways and inhibits NLRP3 activation	Demonstrates anti-inflammatory and protective effects in preclinical models
Senolytic therapies (dasatinib, quercetin, fisetin)	Cellular senescence	Selectively eliminate senescent cells and reduce SASP-mediated inflammation	Effective in aging models; potential strategy to delay gestational tissue aging
SASP-modulating agents (JAK or mTOR inhibitors)	Senescence-associated secretory phenotype	Suppress inflammatory secretory signalling without eliminating senescent cells	May reduce inflammatory amplification in gestational tissues

This table summarizes emerging therapeutic strategies targeting key molecular pathways involved in sPTB within the framework of accelerated gestational aging. These interventions aim to modulate mitochondrial dysfunction, oxidative stress, ferroptosis, inflammasome activation, and cellular senescence, which collectively contribute to premature activation of labour mechanisms. Although most approaches remain in preclinical or early translational stages, they represent promising avenues for the development of targeted prevention strategies for sPTB

ATP: Adenosine triphosphate; IL: Interleukin; JAK: Janus kinase; mTOR: Mechanistic target of rapamycin; NAD⁺: Nicotinamide adenine dinucleotide; NF-κB: Nuclear factor kappa B; NLRP3: NOD-like receptor family pyrin domain containing 3; ROS: Reactive oxygen species; SASP: Senescence-associated secretory phenotype; sPTB: Spontaneous preterm birth

### Precision medicine and personalized interventions

The heterogeneity of sPTB pathogenesis necessitates precision medicine approaches that match interventions to individual pathogenic mechanisms [7]. Molecular profiling of gestational tissues or maternal biomarkers could identify dominant pathogenic pathways like mitochondrial dysfunction, ferroptosis, inflammasome activation, or senescence in guiding selection of targeted therapies [13, 28]. Integration of clinical risk factors, environmental exposures, genetic variants, and molecular biomarkers through machine learning algorithms could enable personalized risk prediction and intervention strategies [56]. The biological heterogeneity of sPTB is further complicated by tissue-specific differences in stress adaptation, inflammatory signalling, and senescence responses across the placenta, fetal membranes, decidua, cervix, and myometrium. Future precision-based therapeutic strategies may therefore require compartment-specific targeting approaches rather than generalized suppression of inflammation or oxidative stress.

### Future research priorities

Key research priorities include longitudinal studies aimed at characterizing the temporal dynamics of mitochondrial dysfunction, oxidative stress, and cellular senescence throughout pregnancy. In parallel, mechanistic investigations are needed to elucidate the complex crosstalk between ferroptosis, mitophagy, inflammasome activation, and EV-mediated signalling pathways that may contribute to premature activation of labour. Future work should also focus on the development and validation of multi-marker biomarker panels capable of improving prediction of sPTB. Additionally, preclinical studies evaluating combination therapies that target multiple pathogenic pathways simultaneously may provide important insights into potential preventive strategies. Clinical trials assessing mitochondria-targeted antioxidants, inflammasome inhibitors, and ferroptosis inhibitors in high-risk pregnancies will be essential for translating these mechanistic insights into therapeutic interventions. Furthermore, investigation of the long-term effects of sPTB prevention strategies on both maternal and offspring health remains an important area of research. Despite growing evidence implicating mitochondrial dysfunction, oxidative stress, ferroptosis, inflammasome activation, and cellular senescence in sPTB, several important limitations remain. Much of the current mechanistic evidence derives from in vitro systems, animal models, or cross-sectional analyses of placental and fetal membrane tissues collected after delivery, thereby limiting the ability to establish temporal sequence or definitive causality in human pregnancy. Furthermore, sPTB likely represents a biologically heterogeneous syndrome rather than a single disease entity, with substantial variability in the relative contribution of inflammatory, metabolic, vascular, environmental, and senescence-associated pathways across different clinical phenotypes. Consequently, several pathways discussed in this review, including cGAS-STING signalling, NAD^+^ metabolic dysfunction, mitophagy dysregulation, EV-mediated senescence propagation, and senolytic therapeutic strategies, should currently be interpreted as emerging mechanistic frameworks requiring further translational validation. Importantly, these pathways are unlikely to function as isolated linear cascades, but rather as dynamically interconnected and tissue-specific stress-response networks operating across the placenta, fetal membranes, decidua, cervix, and myometrium. Future longitudinal multi-omics studies integrating tissue-specific molecular profiling with circulating biomarkers across gestation will therefore be essential to clarify mechanistic hierarchy, identify dominant pathogenic drivers, and distinguish causal pathways from secondary stress responses preceding the clinical onset of labour. Viewed through the framework of accelerated gestational aging, sPTB can be understood as the consequence of converging biological stress pathways, offering a unifying perspective for integrating diverse mechanisms and guiding the development of comprehensive prevention strategies.

## Conclusion

sPTB represents a complex syndrome driven by accelerated gestational aging, wherein mitochondrial dysfunction, oxidative stress, and cellular senescence prematurely activate labour mechanisms. This review has synthesized emerging evidence linking underexplored mechanistic pathways like mitochondrial dynamics and mitophagy, ferroptosis, NAD^+^ metabolism, inflammasome activation, and EV signalling to sPTB pathogenesis. These interconnected processes converge to promote inflammatory signalling, extracellular matrix degradation, and endocrine changes across gestational tissues, coordinated through intercellular communication via DAMPs, cytokines, and EVs.

The accelerated gestational aging framework provides a unifying conceptual model that integrates diverse biological mechanisms and explains the heterogeneity of sPTB. This paradigm shift has important implications for biomarker development and therapeutic strategies. Circulating mtDNA, telomere length, galectin-3, and EV cargo represent promising biomarkers reflecting key pathogenic processes. Multi-marker panels integrating these biomarkers with clinical risk factors may enable personalized risk stratification and targeted interventions.

Therapeutic strategies targeting mitochondrial dysfunction (mitochondria-targeted antioxidants, NAD^+^ precursors), ferroptosis (ferrostatin-1), inflammasome activation (NLRP3 inhibitors, IL-37), and cellular senescence (senolytics) have shown promise in preclinical models and warrant clinical translation. Precision medicine approaches matching interventions to individual pathogenic mechanisms may improve efficacy while minimizing unnecessary treatment.

Future research should focus on elucidating mechanistic crosstalk between pathways, developing validated biomarker panels, and conducting clinical trials of targeted therapies in high-risk pregnancies. Understanding sPTB through the lens of accelerated gestational aging offers hope for developing effective prevention strategies to reduce the global burden of prematurity and improve outcomes for mothers and infants worldwide.

## Data Availability

No datasets were generated or analysed during the current study.

## Abbreviations

A2M: Alpha-2-macroglobulin
ARE: Antioxidant response element
ATP: Adenosine triphosphate
ATP5B: ATP synthase F1 subunit beta
ccf-mtDNA: Circulating cell-free mitochondrial DNA
CD38: Cluster of differentiation 38
cGAS-STING: cyclic GMP-AMP synthase-stimulator of interferon genes
CRISPR/Cas9: Clustered regularly interspaced short palindromic repeats /CRISPR-associated protein 9
DAMP: Damage-associated molecular patterns
DNA: Deoxyribonucleic acid
ER: Endoplasmic reticulum
EV: Extracellular vesicle
FOXO: Forkhead box O
GPX4: Glutathione peroxidase 4
HMGB1: High-mobility group box 1
IL: Interleukin
IRE-1: Inositol-requiring enzyme 1
JAK: Janus kinase
Keap1: Kelch-like ECH-associated protein 1
LHX1: LIM homeobox 1
LINE1: Long interspersed nuclear element-1
lncRNAs: Long non-coding ribonucleic acids
LPS: Lipopolysaccharide
MMP: Matrix metalloproteinase
mtDNA: Mitochondrial DNA
mTOR: Mechanistic target of rapamycin
NAD^+^: Nicotinamide adenine dinucleotide (oxidised form)
NADPH: Nicotinamide adenine dinucleotide phosphate (reduced form)
NF-κB: Nuclear factor- κB
NLRP3: NOD-,LRR- and pyrin domain-containing protein 3
NMN: Nicotinamide mononucleotide
NR: Nicotinamide riboside
Nrf2: Nuclear factor erythroid 2-related factor 2
OXPHOS: Oxidative phosphorylation
p38 MAPK: p38 Mitogen-activated protein kinase
PARPs: Poly(ADP-ribose) polymerases
PGC-1α: Peroxisome proliferator-activated receptor gamma co-activator 1-α
PPROM: Preterm premature rupture of membranes
RGS12: Regulator of G-protein signalling 12
ROS: Reactive oxygen species
SASP: Senescence-associated secretory phenotype
SERPINE1: Serpin family E member 1
SIRT1: Sirtuin 1
SIRT3: Sirtuin 3
SNHG29: Small nucleolar RNS host gene 29
sPTB: Spontaneous preterm birth
TFAM: Mitochondrial transcription factor A
TLR9: Toll-like receptor 9
